# Potent delivery of an MMP inhibitor to the tumor microenvironment with thermosensitive liposomes for the suppression of metastasis and angiogenesis

**DOI:** 10.1038/s41392-019-0054-9

**Published:** 2019-08-09

**Authors:** Yaqi Lyu, Qingqing Xiao, Lifang Yin, Lei Yang, Wei He

**Affiliations:** 1 School of Pharmacy, China Pharmaceutical University, 210009 Nanjing, China; 2 Shanghai Dermatology Hospital, 200443 Shanghai, China

**Keywords:** Metastasis, Drug delivery

## Abstract

Metastasis is a major cause of chemotherapeutic failure and death. Degradation of a specific component of the extracellular matrix (ECM) by matrix metalloproteinases (MMPs) affects the physical barrier of the tumor microenvironment (TME) and induces metastasis. Here, lysolipid-containing thermosensitive liposomes (LTSLs) were prepared to deliver an MMP inhibitor, marimastat (MATT), to the TME to inhibit MMP activity and expression. LTSLs rapidly released their payloads at 42 °C. Compared with the saline control, MATT-LTSLs exhibited enhanced accumulation in the tumor and a 20-fold decrease in tumor growth in 4T1 tumor-bearing mice; moreover, MATT-LTSLs reduced MMP-2 and MMP-9 activity by 50% and 43%, respectively, and downregulated MMP-2 and MMP-9 expression in vivo by 30% and 43%, respectively. Most importantly, MATT-LTSL treatment caused a 7-fold decrease in metastatic lung nodules and a 6-fold reduction in microvessels inside the tumor. We believe this study provides an effective approach for the suppression of metastasis, and the use of a cytotoxic agent in combination with MATT is a potential strategy for metastatic cancer treatment.

## Introduction

The tumor microenvironment (TME) consisting of noncancer cells, extracellular matrix (ECM), blood vessels, and lymphatics is a suitable environment for cancer cells.^1,2^ Critically, ECM composed of various components, such as collagen, laminins, fibronectin, proteoglycans, and glycosaminoglycans^3^ markedly affects tumor initiation, progression, invasion, and migration.^4^ In particular, through the degradation of the components in the ECM by matrix metalloproteinases (MMPs), tumor angiogenesis and metastasis is promoted.^3,5^ Hence, inhibiting the expression and activity of MMPs in the TME is essential to inhibit metastasis and angiogenesis.

More than 10 synthetic MMP inhibitors (MMPIs) have been developed since the 1980s, including batimastat, marimastat (MATT), tanomastat, prinomastat, and rebimastat.^6^ Among the MMPIs, MATT is one of the most successful MMPIs with few side effects; however, its clinical trial was canceled due to not achieving the endpoint of increased survival.^6,7^ MATT, with a low molecular weight of 331.4 Da and solubility of 3.38 g/L, is a broad-spectrum peptidomimetic MMPI that acts by mimicking the substrate of MMPs and working with MMPs in a competitive and reversible manner^8,9^ (Supplementary Table S1). MATT inhibits MMPs with high efficiency and exhibits over 50% MMP inhibition in a nanomolar range.^10^ In particular, the IC_50_ (half-maximal inhibitory concentration) values to inhibit MMP-1, MMP-2, MMP-7, MMP-9, and MMP-14 are 5, 6, 16, 3 and 9 nM, respectively. Indeed, MATT is able to inhibit tumor growth and was a candidate chemotherapeutic agent; however, a clinical study indicated that MATT was no better than placebo at prolonging survival in gastric cancer patients.^11^ Nonetheless, it has been reported that MATT is a promising maintenance treatment following chemotherapy.^12^ Based on the robust inhibitory effect of MATT on MMPs, it is hypothesized that if MATT is efficiently delivered to the TME, this inhibition will enable the maintenance of the TME physical barrier and the resultant suppression of cancer metastasis, as well as angiogenesis.

Nanoscale drug delivery systems can efficiently deliver therapeutics to the disease sites of interest via the enhanced permeation and retention effect and thus have an overwhelming advantage over conventional preparations.^13^ Thus far, approximately 55 nanomedicines have been approved for use in the clinic.^14^ Liposomes, because of their high drug-loading capacity and entrapment capacity, biocompatibility, and safety, have been effectively employed as drug carriers.^15^ Lysolipid-containing thermosensitive liposomes (LTSLs), a type of smart nanocarrier, maintain their integrity at 37 °C (body temperature); however, LTSLs are ruptured during phase transition after heat treatment at 42 °C, which triggers drug release in the local disease region.^16,17^ LTSLs are more effective at delivering chemotherapeutics to the tumor than conventional liposomes due to their increased vascular permeability, the increased tumor accumulation of liposomes, and enhanced drug release into the tumor vasculature and interstitium.^18^ In particular, clinical trials of doxorubicin (DOX)-loaded LTSLs have been approved by the Food Drug Administration for their efficient delivery of DOX to tumor sites, and as a result, LTSLs are promising vehicles for local drug delivery.^19–21^

In this study, LTSLs were prepared for local delivery of MATT to the TME, with the aim of inhibiting breast cancer metastasis. To obtain proof of the concept, various experiments, including western blotting, gelatin zymography, determination of angiogenesis, and lung metastasis, were carried out. Significantly, LTSLs delivered MATT to the TME with high efficiency and suppressed lung metastasis and angiogenesis in 4T1-bearing mice, as well as inhibiting MMPs. Compared with the inhibition of tumor growth, MATT, particularly MATT-LTSLs, was more efficient at inhibiting angiogenesis and metastasis. We believe that this finding provides a novel strategy for adjuvant chemotherapy for metastatic cancer.

## Results

### Preparation and characterization of LTSLs

**Fig. 1:**
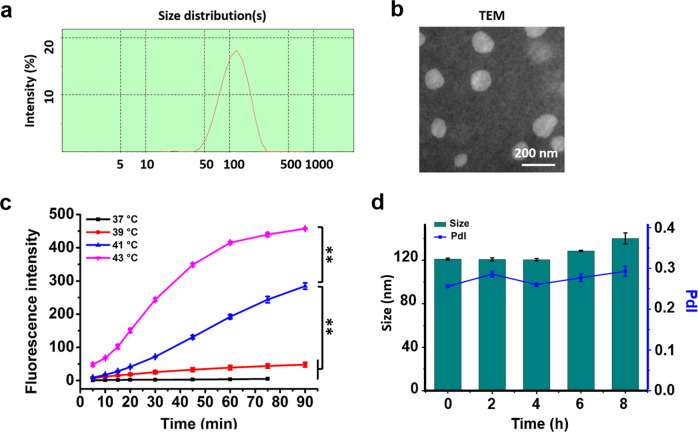
Characterization of the MATT-LTSLs. **a** Size distribution and **b** TEM image of LTSLs. **c** Thermosensitivity of CF-LTSLs at different temperatures. **d** Stability of the MATT-LTSLs in serum (mean ± SD, *n* = 3, **p < 0.01)

MATT-LTSLs were prepared by a film hydration method followed by a probe ultrasonic treatment after the addition of MATT solution, along with lipid composition of dipalmitoylphosphatidylcholine (DPPC)/1-stearoyl-2-hydroxy-*sn*-glycerol-3-phosphocholine (1-StePc)/1,2-distearoyl-*sn*-glycero-3-phosphoethanolamine-*N*-[methoxy(polyethylene glycol)-2000] (DSPE-PEG2000) at 86:10:4 (mass ratio). A high-performance liquid chromatography (HPLC) assay revealed that the entrapment efficiency of MATT was 56% and the drug-loading (DL) rate was 2.8%. Determination by dynamic light scattering (DLS) revealed that MATT-LTSLs had a diameter of approximately 100 nm with a polydispersity index (PdI) <0.3 (Fig. 1a). The transmission electron microscopy (TEM) examination revealed spherical particles of 90–100 nm diameter (Fig. 1b), which is consistent with the DLS result.

### In vitro drug release

To detect the thermosensitivity of LTSLs, a test of drug release at different temperatures was performed, in which a fluorophore, 5(6)-carboxyfluorescein (CF), was encapsulated. The properties of CF are shown in Supplementary Table S1. As displayed in Fig. 1c, raising the temperature from 37 to 43 °C increased the drug release rate; and especially, at 43 °C, CF-LTSLs displayed extremely fast release compared with that at 37 °C (normal body temperature). This temperature sensitivity is due to the presence of lysolipids in the lipid bilayer of LTSLs, which significantly disturb the ordered phospholipid arrangement at 42 °C.^18,22^ These data implied that MATT would be well encapsulated in LTSLs following injection into blood circulation, while rapid drug exposure at the local disease site would occur post treatment at 42 °C.

### Stability

The stability of nanoparticles in serum is critical for their targeting to the disease site.^23^ The stability of LTSLs was studied by measuring the changes in particle size and PdI using the DLS method. After being stored in 10% fetal bovine serum (FBS) medium for 8 h at 37 °C, the particle sizes of MATT-LTSLs and PdI were not altered (Fig. 1d), indicating that MATT-LTSLs were stable at 37 °C.

### In vitro cytotoxicity

Cytotoxicity against the 4T1 and MDA-MB-435 human breast cancer cell lines was determined by a 3-(4,5-dimethylthiazol-2-yl)-2,5-diphenyltetrazolium bromide (MTT) assay with a 48-h incubation at 37 °C. LTSLs without payload generated little toxicity to 4T1 and MDA-MB-435 cells, even at high concentrations of lipids (Supplementary Fig. S1c). The effect of the drug-loading formulations, free MATT and MATT-LTSLs, on cytotoxicity in 4T1 and MDA-MB-435 cells is shown in Supplementary Fig. S1a, b. Free MATT showed minimal toxicity against these two cell lines, despite the concentration difference. Additionally, MATT-LTSLs did not affect the viability at MATT concentrations <10 µg/mL for MDA-MB-435 cells and 5 µg/mL for 4T1 cells; however, at higher concentrations of 50 µg/mL, MATT-LTSLs were toxic to these cell lines, with cell viability <35% and 15% for 4T1 cells and MDA-MB-435 cells, respectively. Additionally, hyperthermia (HT) treatment at 42 °C did not influence the cytotoxicity. MMPs, particularly MT1-MMP, are multifunctional proteases that regulate ECM degradation, pro-MMP-2 activation, and viability; importantly, the cytoplasmic domain of MT1-MMP is required for cell survival.^24^ Here, the improved uptake of MATT by LTSLs might result in increased binding of MATT with the cytoplasmic domain of MT1-MMP, which subsequently affects cell viability.

### In vitro inhibition of MMPs

**Fig. 2:**
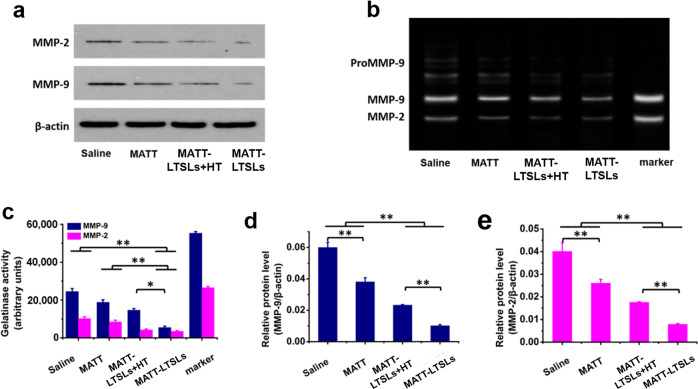
MMP inhibition in vitro in 4T1 cells. **a** Western blot analysis of MMP-2 and MMP-9 expression. **b** Gelatin zymography of MMP-2 and MMP-9 activity. **c** Quantitative analysis of MMP activity from zymograms using a computer analysis program. Quantitative analysis of **d** MMP-9 and **e** MMP-2 expression. β-Actin was used as a loading control (mean ± SD, *n* = 3, *p < 0.05 and **p < 0.01)

The in vitro expression and activity of MMP-2 and MMP-9 in 4T1 cells was assayed by western blot and gelatin zymography after incubation with MATT-LTSLs for 12 h followed by mild HT treatment at 42 °C or not. The western blot analysis demonstrated that the expression of MMPs in cells treated with MATT-LTSLs and MATT-LTSLs + HT was significantly downregulated compared with that of cells treated with saline and free MATT (Fig. 2a, d, e). A further comparison revealed that the MMP expression in MATT-LTSL-treated cells was lower than that of MATT-LTSL + HT-treated cells.

In the gelatin zymography analysis performed by analyzing protein extracts from treated cells by sodium dodecyl sulfate-polyacrylamide gel electrophoresis (SDS-PAGE) containing 0.4% gelatin, bright gel bands indicating MMP activity appear as the gelatin is degraded by the MMPs.^25^ In the saline group, bright bands indicating high MMP-2 and MMP-9 activity levels were evident. Treatment with MATT-LTSLs or MATT-LTSLs + HT decreased the area and brightness of the bands more significantly compared to treatment with free MATT (Fig. 2b, c). Again, MATT-LTSLs exhibited a more profound reduction in MMP activity than MATT-LTSLs + HT. Generally, MMPs in the TME are synthesized and secreted by tumor cells; thus, the in vitro inhibition of MMP expression and activity is effectively reduced by the improved cellular uptake of the MMPI, MATT, via MATT-LTSLs. Collectively, our results indicate that both MATT-LTSLs and MATT-LTSLs + HT are capable of decreasing the expression and activity of MMP-2 and MMP-9 in 4T1 cells, with the former showing enhanced inhibition.

### In vivo tumor targeting and biodistribution

**Fig. 3:**
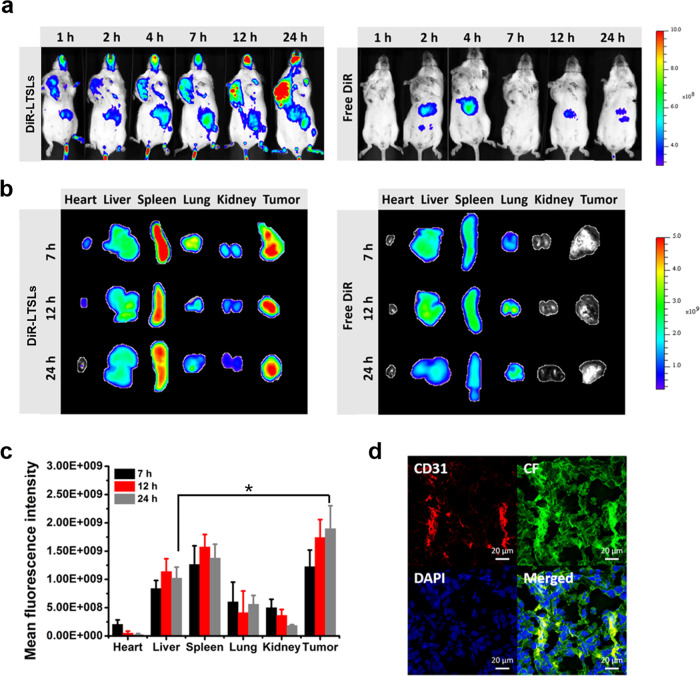
Biodistribution of DiR-labeled LTSLs in 4T1 tumor-bearing Balb/C mice. **a** Image of the whole body at different time points after administration of DiR-LTSLs or free DiR through the tail vein (*n* = 3) at a DiR dose of 0.5 mg/kg. **b** Image of the major tissues collected at 7, 12, and 24 h post injection. **c** Ex vivo 24-h quantification of DiR-LTSLs in different tissues (mean ± SEM, *n* = 3, *p < 0.05). **d** Intratumoral distribution of CF-LTSLs imaged by confocal laser scanning microscopy (CLSM). The microvessels were stained with a Cy7-labeled anti-CD31 antibody (red), and the nucleus was stained with 4′,6-diamidino-2-phenylindole (DAPI) (red: CD31; green: CF; and blue: DAPI)

Following the injection of DiR-LTSLs, in vivo imaging of 4T1 tumor-bearing mice at specific time points was performed. An increase in fluorescence in the tumor over time was shown (Fig. 3a); dramatic fluorescence appeared at 24 h. To further determine the biodistribution of the DiR-LTSLs, the major tissues were collected at 7, 12, and 24 h post injection. The robust fluorescence observed in the isolated tumor was equivalent to that in the spleen, demonstrating the efficient accumulation of DiR-LTSLs in the tumor (Fig. 3b, c).

To study the penetration of LTSLs into the tumor, the tumors were collected to prepare frozen tumor sections for microvessel staining with a Cy7-labeled anti-CD31 antibody (red) at 24 h after injection of CF-LTSLs into the 4T1 tumor-bearing mice. Strong yellow fluorescence was observed in the merged image, which indicated the colocalization of CF-LTSLs with microvessels inside the tumors and demonstrated that CF-LTSLs could penetrate the tumor (Fig. 3d).

### In vivo antitumor efficacy

**Fig. 4:**
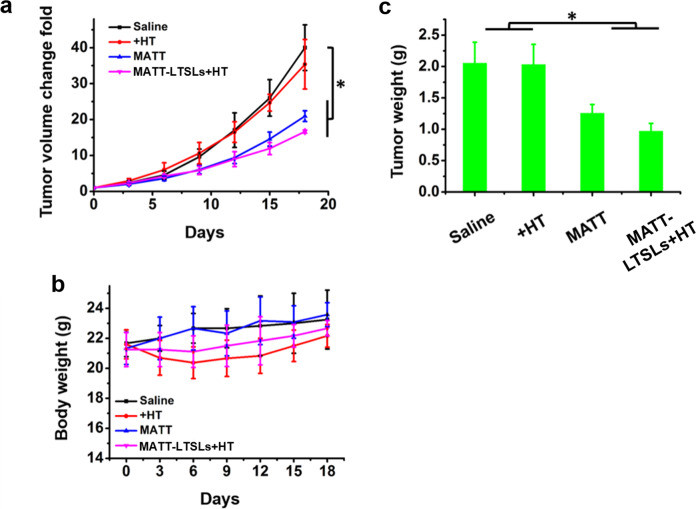
In vivo antitumor efficacy in 4T1 tumor-bearing Balb/C mice. Free MATT, MATT-LTSLs, and the same volume of saline were injected via the tail vein every 3 days at a MATT dose of 5 mg/kg. **a** Tumor volume fold change. **b** Body weight changes. **c** Tumor weight. The tumor was weighed on day 19 post injection (mean ± SD, *n* = 6, *p < 0.05)

To investigate the antitumor activity, free MATT and MATT-LTSLs were administered to 4T1 tumor-bearing mice every 3 days for a duration of 18 days. The groups treated with saline or HT did not display inhibition of tumor growth, with a more than 35-fold increase in tumor volume at day 18 after administration (Fig. 4a). The MATT treatment led to a 20-fold increase in tumor volume at day 18, while MATT-LTSLs + HT treatment resulted in an approximately 15-fold increase, demonstrating that MATT-LTSLs + HT treatment improved the inhibition of tumor growth. This inhibition of tumor growth was confirmed by the measurement of tumor weight at day 18 (Fig. 4c). No significant body weight loss was observed in the MATT-LTSL group compared with the saline group, which demonstrated the safety of this formulation (Fig. 4b).

To further study the apoptosis and proliferation of cancer cells in the tumor, the tumors were harvested at the end of the experiment and cut into 5-µm sections that were stained with hematoxylin and eosin (H&E), TUNEL, and Ki67. These results are depicted in Supplementary Fig. S2. The H&E analysis of the various treatment groups indicated the lowest number of cancer cells in the MATT-LTSLs + HT group mice (Supplementary Fig. S2a). Accordingly, the terminal deoxynucleotidyl transferase dUTP nick-end labeling (TUNEL) and Ki67 assays demonstrated the highest number of positive cells after treatment with MATT-LTSLs + HT compared with that of the other formulation treatments, which was confirmed by the quantitative analysis (Supplementary Fig. S2b, c). Taken together, these data indicate that compared to free MATT, MATT-LTSLs + HT had enhanced antitumor efficacy.

### Inhibition of angiogenesis and lung metastasis

**Fig. 5:**
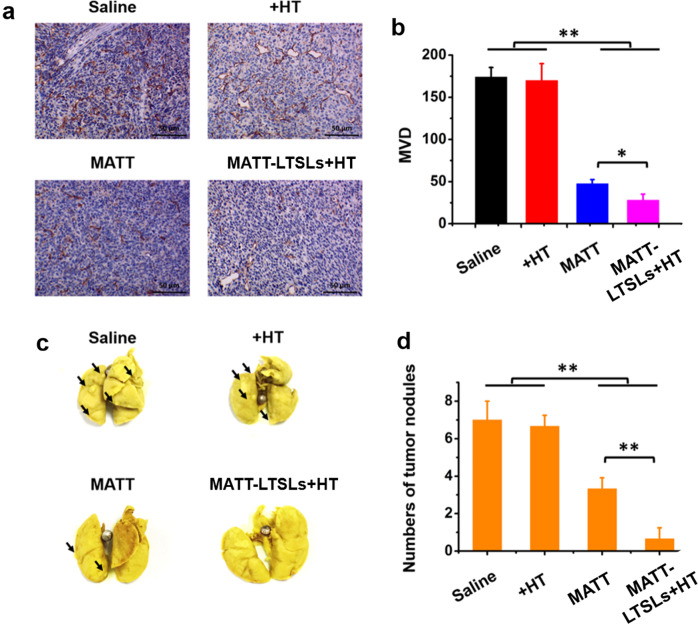
Inhibition of angiogenesis and lung metastasis. **a** Immunochemistry of CD31 staining for microvessels in tumor tissues collected from 4T1 tumor-bearing Balb/C mice at day 19 after treatment. **b** Quantitative analysis of MVD. MVD was quantified in five representative fields of cell nuclei under an optical microscope. **c** Digital images of lungs collected from 4T1 tumor-bearing Balb/C mice at day 19 after treatment. The arrow indicates the tumor nodules in the lungs. **d** Quantitative analysis of tumor nodules in the lungs by counting the number of nodules (mean ± SD, *n* *=* 3, *p < 0.05 and **p < 0.01)

ECM degradation by MMPs results in invasive growth, metastasis, and angiogenesis of cancer and various chronic inflammatory diseases.^26^ Here, an examination of angiogenesis and lung metastasis inhibition was performed at the end of the treatment. Angiogenesis was assessed by analyzing the microvascular density (MVD) in the sectioned tumors stained with an anti-CD31 antibody. In the control groups (saline and HT), the brown-stained microvessels stained inside the tumor were easily observed with MVD with up to 170 vessels/field (Fig. 5a, b). MATT treatment reduced the number of microvessels >3-fold compared with the controls. Most critically, MATT-LTSLs + HT treatment decreased the MVD by approximately 7-fold and 1-fold compared with that of the control and free drug treatments, respectively. These results demonstrate that MATT, especially MATT-LTSLs + HT, produced a profound antiangiogenic effect.

The 4T1 cell line is derived from a highly metastatic breast cancer and mainly metastasizes to the lung and liver.^27^ Hence, the metastasis of the tumor to the lung in 4T1 tumor-bearing mice was tested at day 19 post treatment by quantifying the colonies that appeared in the lung tissue. As displayed in Fig. 5c, a number of metastatic nodules of considerable size were observed in the saline and HT groups, with a mean number of seven nodules per lung (Fig. 5d). In contrast, the treatment with MATT or MATT-LTSLs + HT induced a 1-fold and 6-fold reduction in the metastatic nodules, respectively, and the comparison between the MATT-LTSLs + HT and MATT groups showed more than 6-fold decrease in tumor nodules in the MATT-LTSLs + HT group. Overall, free MATT treatment significantly inhibited cancer metastasis in 4T1 tumor-bearing mice, and importantly, MATT-LTSLs + HT treatment dramatically enhanced this inhibition.

### In vivo inhibition of MMPs

**Fig. 6:**
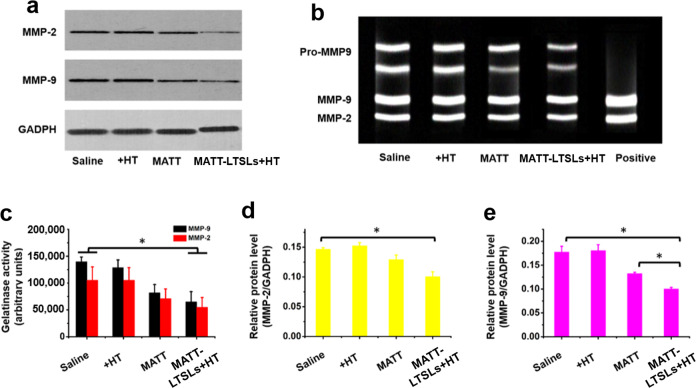
MMP inhibition in vivo. **a** Western blot analysis of MMP-2 and MMP-9 expression in tumors. **b** The activity of MMP-2 and MMP-9 measured by gelatin zymography. **c** Quantitative analysis of MMP activity from zymograms using a computer analysis program. Quantitative analysis of **d** MMP-2 and **e** MMP-9 expression. Glyceraldehyde 3-phosphate dehydrogenase (GADPH) was used as a loading control. Tumors used in these experiments were collected from 4T1 tumor-bearing Balb/C mice on day 19 after treatment (mean ± SD, *n* = 3, *p < 0.05)

Because of the ECM degradation by tumors, MMPs, particularly MMP-2 and MMP-9, play an essential role in the angiogenesis and metastasis of cancer. To examine the activity and expression of MMP-2 and MMP-9 in the tumor, the tumors were harvested from 4T1 tumor-bearing mice at day 18 after treatment for gelatin zymography and western blot analysis. First, the activity of MMP-2 and MMP-9 was assayed by gelatin zymography in which bright gel bands indicating the MMP activity appear after gelatin degradation by MMPs.^25^ As depicted in Fig. 6b, c, significant bright bands were visualized in the saline- and HT-treated groups, while the MATT and MATT-LTSLs + HT groups exhibited weak bands in terms of area and brightness. Further quantitative analysis demonstrated that MATT and MATT-LTSLs + HT treatment reduced the MMP-2 activity by approximately 25% and 50%, respectively, and decreased the MMP-9 activity by approximately 43% and 57%, respectively, compared with saline treatment. These results indicate that the in vivo activity of MMP-2 and MMP-9 was significantly inhibited by MATT treatment, especially MATT-LTSLs + HT treatment.

MMP-2 and MMP-9 expression in the tumor was further detected by a western blot assay (Fig. 6a, d e). Compared with the treatment with saline or HT, treatment with MATT or MATT-LTSLs + HT efficiently downregulated the expression of MMP-2 and MMP-9 (Fig. 6a). Specifically, free MATT treatment decreased the expression of MMP-2 and MMP-9 by 17% and 25%, respectively, while MATT-LTSLs + HT treatment reduced their expression levels by 30% and 43%, respectively, implying significantly improved inhibition of MMP expression. Overall, MATT-LTSLs + HT treatment enabled the effective downregulation of MMPs (Fig. 6d, e).

## Discussion

MATT exhibited modest efficacy in delaying cancer progression in preclinical and clinical studies. Furthermore, a phase III study on MATT was terminated for cumulative toxicity due to inflammation and musculoskeletal pain.^10,28^ However, it has also been reported that certain cancer patients, in particular, those who received chemotherapy in advance, benefited from maintenance treatment with MATT.^29^ In this study, the MATT-LTSLs + HT group exhibited 15-fold tumor growth vs. 35-fold growth exhibited by the saline treatment in 4T1 tumor-bearing mice, demonstrating that LTSLs are a promising carrier for improved MATT antitumor efficacy. Nonetheless, due to MATT’s low cytotoxicity against cancer cells (Supplementary Fig. S1), its antitumor activity, even when efficiently delivered to the tumor, is modest compared with that of a robust cytotoxic agent such as paclitaxel.^30^

LTSLs efficiently deliver MATT to the tumor location and enhance the in vivo inhibition of MMPs. Gelatin zymography and western blot analysis showed that compared with free MATT, MATT-LTSLs significantly enhanced the inhibition of MMP-2 and MMP-9 activity and expression. These results stem from the tumor-targeting ability of LTSLs, showing that LTSLs not only were able to accumulate in tumors but also were able to penetrate the tumors (Fig. 3). Subsequently, the penetrated LTSLs rapidly released their payloads within the microvessels inside the tumor induced by local HT treatment of the tumors, resulting in the increased localization of the payload in the entire tumor.^20^ Indeed, the temperature sensitivity of the LTSLs was confirmed by the in vitro release test.

Compared with its ability to inhibit tumor growth, MATT is a more potent inhibitor of angiogenesis and metastasis, and importantly, these effects are markedly enhanced when LTSLs are used as MATT carriers. Since the discovery of MATT, a considerable number of studies have focused on its antitumor efficacy regarding the inhibition of tumor growth rather than its suppression of cancer metastasis and angiogenesis. Essentially, the main function of MATT is to inhibit the MMPs in the TME instead of killing cancer cells directly, assisting the maintenance of the physical barrier of the TME and reducing the metastasis. Here, MATT-LTSLs enabled a 7-fold decrease in the number of metastatic nodules in the lung and a 6-fold reduction in the number of microvessels inside the tumor, which implies profound antimetastatic and antiangiogenic effects. Additionally, free MATT exhibited a 3-fold and 1-fold decrease in metastasis and angiogenesis. These results also demonstrate that only MATT is efficiently delivered to the TME. These two inhibitory effects are greatly enhanced because LTSLs deliver MATT directly to the tumor and subsequently inhibit MMP activity and expression, prevent ECM degradation, and protect the TME from destruction, ultimately restricting the cancer cells to the TME. Once metastasis has been established, chemotherapy always fails to induce a durable response, ultimately resulting in cancer patient death.^27^ Therefore, our study provides a competent strategy for inhibiting metastasis. Undeniably, the use of MATT-LTSLs alone is not efficient in killing cancer cells due to MATT’s low toxicity to cells; consequently, the combined use of a cytotoxic agent such as paclitaxel with MATT is a promising treatment approach for metastatic cancer. Further study is currently underway.

In this study, LTSLs with excellent tumor-targeting ability enabled the efficient delivery of MATT to the tumor location, resulting in the inhibition of MMP activity and expression. MATT-LTSLs have a modest effect on the suppression of tumor growth due to MATT’s low cytotoxicity against cancer cells. However, this formulation has significant antiangiogenesis and antimetastasis effects. We believe this study offers a potent strategy for suppressing metastasis and the use of a cytotoxic agent in combination with MATT is a potential strategy for treating metastatic cancer. Taken together, our results indicate that by delivering MATT to the tumor site with LTSLs, cancer metastasis and angiogenesis are markedly inhibited due to the suppression of the activity and expression of MMPs in the TME.

## Methods

### Preparation and characterization

#### Preparation of MATT-LTSLs

The MATT-LTSL lipids were composed of DPPC:1-StePc:DSPE-PEG2000 at a mass ratio of 86:10:4. These phospholipids were first dissolved in a 3:1 (v/v) solution of chloroform and then dried under vacuum at 45 °C. Next, a solution of pH 6.5 phosphate-buffered saline (PBS) containing MATT was used to hydrate the lipid film at 45 °C for 40 min. Then, the raw liposome solution was treated by an ultrasonic probe and extruded through a membrane filter with a pore size of 0.22 μm. Finally, the preparation was dialyzed (MWCO 8000–14,000) against pH 6.5 PBS to remove the free MATT. CF- and DiR-loaded LTSLs were prepared with the same method.

#### Particle size and zeta potential

Size, PdI, and zeta potential were measured by DLS using a Malvern Zetasizer (Brookhaven Instruments, NY, USA).

#### Transmission electron microscopy

Morphology examination was performed on a JEM-1230 TEM (Tokyo, Japan) at 200 kV. The sample was dropped on a carbon mesh, stained with 2% (w/v) phosphotungstic acid for 10 min and dried at room temperature.

#### DL% and EE%

After being purified through dialysis, MATT-LTSLs were collected and lysed with 5 mg/mL SDS. The EE% and DL% of MATT was determined by an HPLC method. The DL% and EE% was calculated as follows:

$${\mathrm{EE\% }} = \frac{{{\mathrm{Amount}}\,{\mathrm{of}}\,{\mathrm{MATT}}\,{\mathrm{entrapped}}}}{{{\mathrm{Amount}}\,{\mathrm{of}}\,{\mathrm{MATT}}\,{\mathrm{added}}}} \times 100{\mathrm{\% }},$$

$${\mathrm{DL\% }} = \frac{{{\mathrm{Amount}}\,{\mathrm{of}}\,{\mathrm{MATT}}\,{\mathrm{entrapped}}}}{{{\mathrm{Amount}}\,{\mathrm{of}}\,{\mathrm{lipids}}}}100{\mathrm{\% }}.$$

The drug content was assayed on an LC-10AT HPLC system (Shimadzu, Japan). The separation was conducted on an ODS C18 column (4.6 mm × 250 mm, Diamonsil, China) at 30 °C with a mobile phase of methanol/H_2_O (65/35, v/v, pH = 3) at a flow rate of 1 mL/min at 210 nm.

### In vitro drug release

CF was chosen as a model compound to study the temperature sensitivity features of LTSLs. The test was carried out through a dialysis method in a release tester (ZRS-8G, Tianjin, China). Briefly, 1.5 mL of CF-LTSLs was added to a dialysis bag (MWCO 8000–14,000) and incubated in 100 mL of pH 6.5 PBS at different temperatures for 90 min. The release profile was determined by measuring the fluorescence intensity of CF using a fluorescence spectrometer (RF-5301PC, Shimadzu, Japan) at excitation and emission wavelengths of 492 and 515 nm, respectively.

### Stability

One milliliter of nanoparticle suspension was mixed with 4 mL of 1640 medium containing 10% FBS and incubated at 37 °C. At specific time intervals, the samples were withdrawn and tested by DLS (Brookhaven Instruments, Holtsville, NY, USA).

### Cytotoxicity

Cell viability was measured by an MTT assay. Briefly, cells were seeded in 96-well plates at a density of 5 × 10^3^ cells/well and cultured for 48 h at 37 °C. Then, the cells were cultured with fresh medium containing different formulations along with HT treatment for another 48 h. For the MATT-LTSLs + HT group, MATT-LTSLs were incubated for 60 min at 42 °C before being used to treat the cells Subsequently, the cells were incubated with 20 μL of MTT (5 mg/mL) for 4 h or 150 μL of dimethyl sulfoxide. The absorbance of each well was measured at 570 nm using a microplate reader (Multiskan FC, Thermo Fisher Scientific, USA).

### In vitro inhibition of MMPs

#### Western blot analysis

First, the cells were incubated in RIPA buffer on ice for 10–20 min, followed by homogenization and centrifugation for 10 min at 9000 × *g* at 25 °C. The total protein was measured using a BCA protein assay kit (Beyotime, China) and separated by 10% SDS-PAGE. Then, the protein was transferred to a nitrocellulose membrane and incubated in a blocking solution containing 5% skim milk powder at 37 °C for 2 h. After being rinsed with PBS containing Tween-20 (PBST) 2–3 times, the membrane was incubated with primary antibody at 4 °C for 2 h and a secondary antibody at 4 °C overnight. Next, the target proteins were detected by staining with a chemiluminescence kit (KeyGEN Biotech., China), and blot images were acquired using an Odyssey Infrared Imaging System (LICOR Biotechnology, Lincoln, NE, USA). β-Actin served as an internal control to normalize the protein expression levels. The integrated optical density was calculated using the gel optical density analysis software Gel-Pro 4.0.

#### Gelatin zymography

Briefly, 10 μL of protein extract from treated cells was analyzed by SDS-PAGE containing 0.4% gelatin. After being rinsed with 2.5% Triton X-100, the gels were incubated in 8% separation solution for 4 h at room temperature, followed by staining with Coomassie blue for 2 h, and destaining with 25% methanol and 10% acetic acid for 1 h. Clear bands on the dark background indicated gelatin degradation. The quantitative analysis of gelatinase activity from the zymography was conducted by Gel-Pro 4.0.

### Biodistribution study

The animals used in this experiment received care in compliance with the Principles of Laboratory Animal Care and the Guide for the Care and Use of Laboratory Animals. All animal experiments were performed in accordance with the protocol approved by the China Pharmaceutical University Institutional Animal Care and Use Committee. First, 4T1 cells suspended at a density of 1 × 10^7^ cells/mL were injected into the upper dorsal region of Balb/C mice (females, 18–20 g) subcutaneously in a volume of 0.1 mL for each mouse. When the tumor volume reached 500 mm^3^, free DiR and DiR-LTSLs were injected into the tumor-bearing mice via the tail vein at a fixed DiR dose of 0.5 mg/kg. At 1, 2, 4, 7, 12, and 24 h post injection, the treated mice were anesthetized with isoflurane to detect DiR fluorescence using an In Vivo Imaging System (IVIS Spectrum, Perkin-Elmer, USA). At 7, 12, and 24 h post injection, the mice were sacrificed to collect the major tissues for ex vivo fluorescence imaging.

### In vivo antitumor efficacy

#### Antitumor activity

The 4T1 tumor-bearing mice were divided into four groups (*n* = 6), which were injected with 0.2 mL of free MATT or MATT-LTSLs via the tail vein every 3 days at a MATT dose of 5 mg/kg. Following anesthesia with 10% chloral hydrate (w/v) at a dose of 400 mg/kg, HT treatment was performed by incubating the tumor inside a water bath at 42 °C for 45 min. The tumor volume and body weight were calculated every 3 days. At the end of the treatment, the mice were sacrificed to harvest the main organs for further analyses.

#### Apoptosis, proliferation, and histological analysis

Apoptosis and proliferation in the tumor was determined by TUNEL and Ki67 immunochemistry, respectively. Collected tumors were fixed in 4% paraformaldehyde, embedded in paraffin, and cut into 5-μm-thick sections. Then, TUNEL and Ki67 immunochemistry were performed according to the standard instructions. The sections were examined using an optical microscope (B1-330, Motic, China) and quantified in five representative fields. For histological analysis, the paraffin section was stained with H&E and observed under an optical microscope for pathological analysis.

### Inhibition of angiogenesis and lung metastasis

#### Inhibition of angiogenesis

The tumor sections (5 μm) were prepared for immunohistochemistry as previously described. First, sections were preincubated in 3% hydrogen peroxide in methanol for 15 min at room temperature. Then, sections were treated with 0.25% trypsin for 10 min, stained with a primary anti-CD31 monoclonal antibody overnight and a secondary antibody for 30 min, incubated with fresh DAB, stained with hematoxylin for 10 min, and finally observed under an optical microscope. MVD was calculated in five representative fields.

#### Inhibition of lung metastasis

At day 19 post administration, mice in each group were sacrificed, and the lungs were collected. For the detection of lung metastasis, the collected lungs were first fixed in Bouin’s solution for 72 h at room temperature; subsequently, the number of tumor nodules apparent in the lungs was calculated to assess the metastasis of the breast cancer to the lung.

#### In vivo inhibition of MMPs

At day 19 after treatment, the mice in each group were sacrificed for the collection of the tumors. Western blotting and gelatin zymography were conducted as described above.

### Statistical analysis

The data were expressed as the mean ± SD. The statistical significance between groups was assessed by one-way *t* test analysis. Significance was considered as a *p* <0.05).

## Supplementary information

SUPPLEMENTAL MATERIAL
