# Supplementary material for: Potent delivery of an MMP inhibitor to the tumor microenvironment with thermosensitive liposomes for the suppression of metastasis and angiogenesis
Source: Signal Transduct Target Ther. 2019 Aug 9;4:26. doi: 10.1038/s41392-019-0054-9 (PMC6799847; doi:10.1038/s41392-019-0054-9)
Supplement: Supplementary file 1 — SUPPLEMENTAL MATERIAL [file 41392_2019_54_MOESM1_ESM.docx]

**Supplementary materials**

**Potent delivery of MMP inhibitor to the tumor microenvironment with thermosensitive liposomes for** **anti-metastasis and -angiogenesis**

Yaqi Lyu ^a^, Qingqing Xiao ^a^, Lifang Yin ^a^, Lei Yang*^, a^, Wei He ***^, b, a^**

*^a^ School of Pharmacy, China Pharmaceutical University, Nanjing 210009, P.R. China*

*^b^ Shanghai Dermatology Hospital, Shanghai 200443, PR China*

**^*^**Corresponding authors: Lei Yang and Wei He

E-mail address: yangleisypu@126.com (L., Yang), weihe@cpu.edu.cn (W., He)

*Materials*

MATT was purchased from Nanjing Adooq Co., Ltd. (Jiangsu, China). dipalmitoyl phosphatidylcholine (DPPC) and 1,2-distearoyl-sn-glycero-3-phosphoethanolamine-N-[methoxy(polyethylene glycol)-2000] (DSPE-PEG2000) were purchased from Lipoid (Ludwigshafen, Germany). 1-stearoyl-2-hydroxy-sn-glycero-3-phosphocholine (1-StePc) was purchased from Shanghai AVT Pharmaceutical Technology Co., Ltd. (Shanghai, China). 5(6)-Carboxyfluorescein (CF) and 3-(4,5-dimethylthiazol-2-yl)-2,5-diphenyltetrazolium bromide (MTT) were purchased from Sigma Aldrich (St Louis, MO, USA). AnnexinV-FITC/PI Apoptosis Kit was obtained from Nanjing KeyGEN Biotech Co., Ltd. (Nanjing, China). Fetal bovine serum, RPMI-1640, DMEM, trypsin and antibiotics were from Nanjing Wisent Biotechnology Co., Ltd. (Jiangsu, China). 1,1`-dioctadecyl-3,3,3`,3`-tetramethylindotricarbocyanine iodide (DiR) was purchased from Biotium, Inc. (Hayward, CA, USA). Cy7-labeled anti-CD31 antibody was from Abcam (Cambridge, UK). H&E staining, TUNEL and Ki67 kits were purchased from Beyotime Institute of Biotechnology (Haimen, China). The mouse breast carcinoma cell line 4T1 and human breast carcinoma cell line MDA-MB-435 were purchased from Nanjing KeyGEN Biotech Co., Ltd. (Nanjing, China). All chemicals and reagents were of analytical grade.


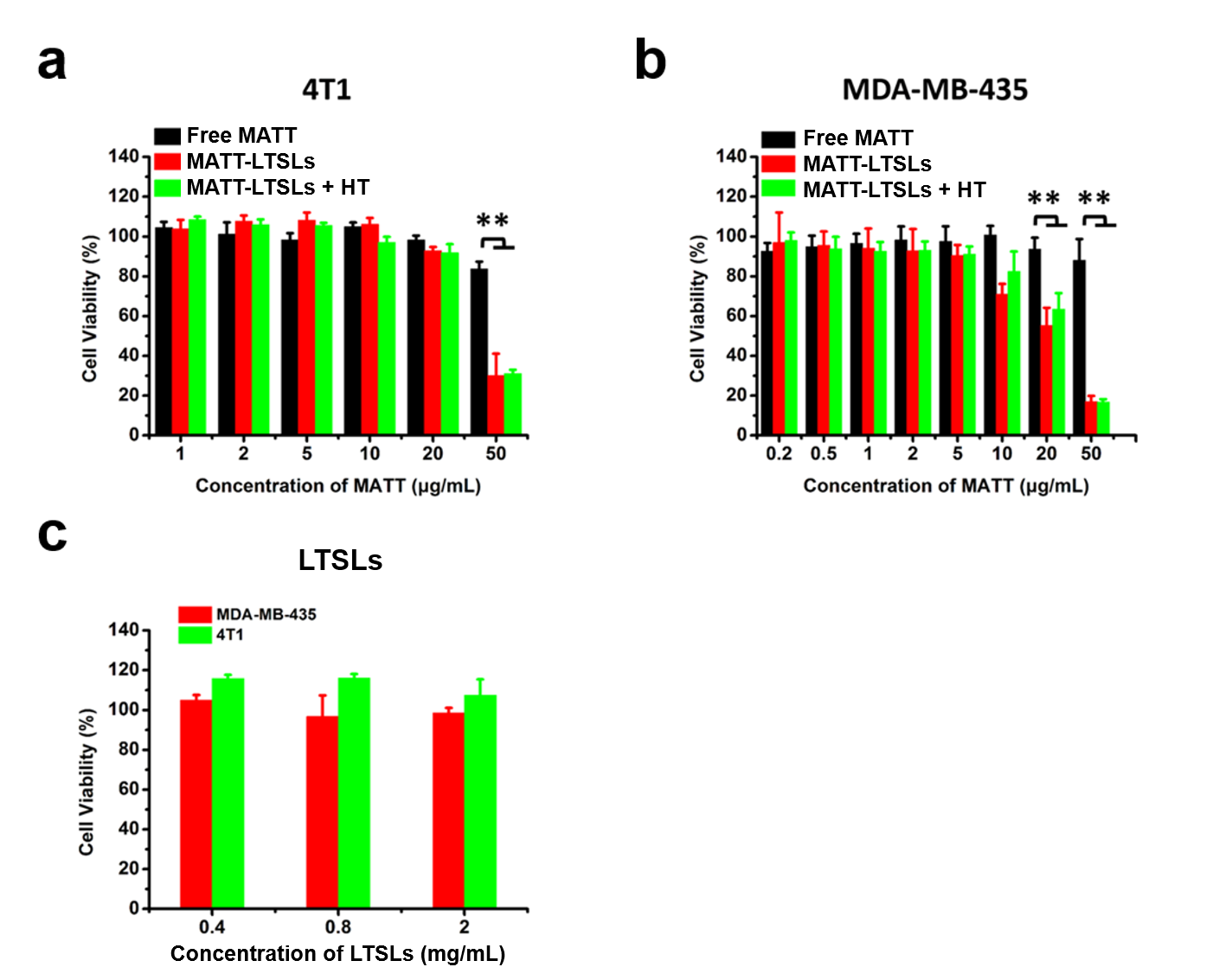


**Figure S1.** Cytotoxic study. Effect of different formulations loading MATT on viability of (a) 4T1 or (b) MDA-MB-435 cells. (c) Cytotoxicity of blank LTSLs in 4T1 and MDA-MB-435 cell lines. (mean ± S.D., *n* = 5, ***p* < 0.01).


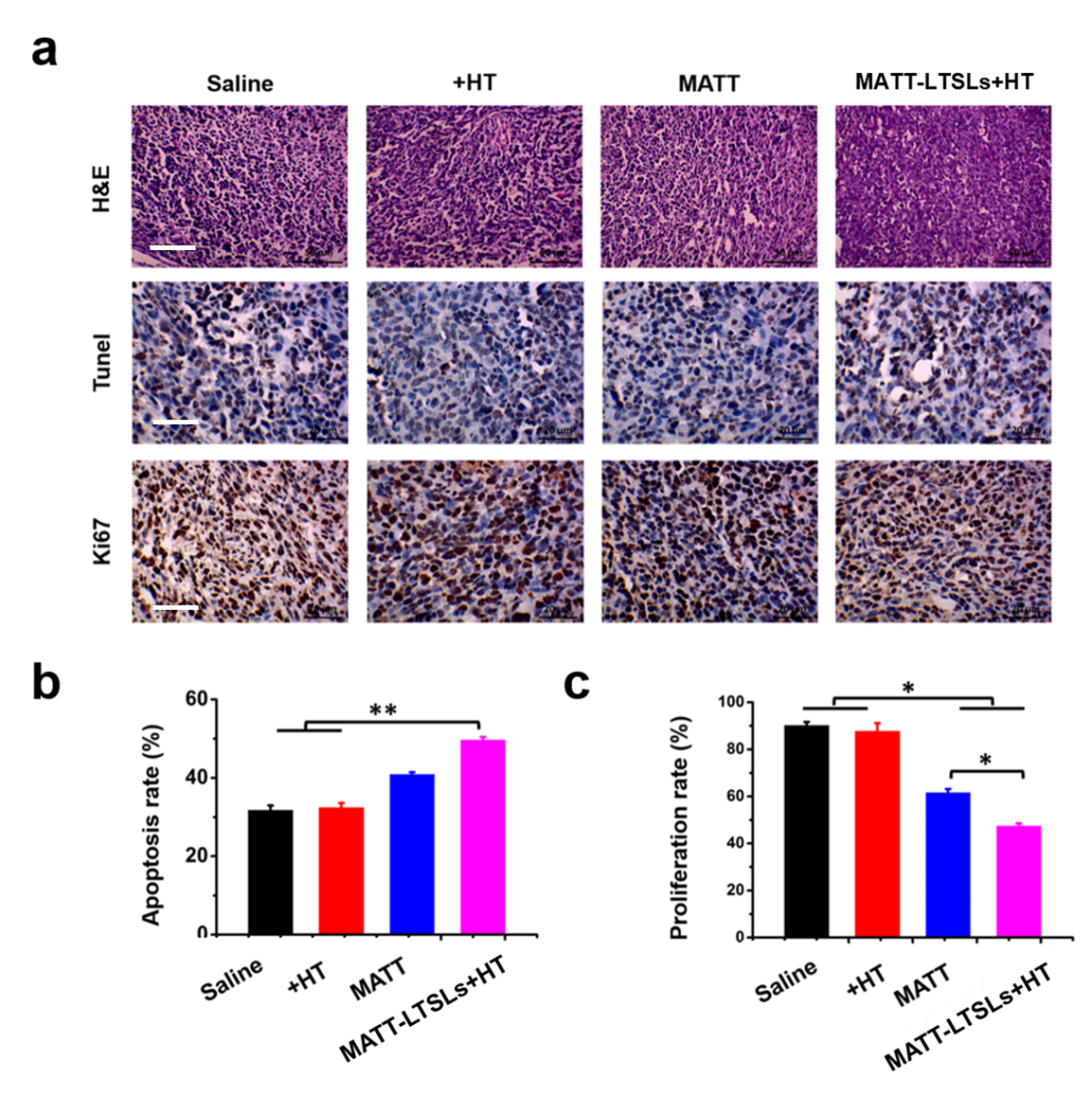


**Figure S2.** Histological study. (a) H&E, TUNEL and Ki67 staining of tumor sections collected from 4T1 tumor-bearing Balb/C mice on day 19 after treatment. The scale bar is 20 µm. Quantitative analysis of (b) apoptosis rate and (c) proliferation rate. Cell apoptosis and proliferation rate were quantified by five representative fields of cell nuclei under an optical microscope. (mean ± S.D., *n* = 3, **p* < 0.05, ***p* < 0.01).

**Table S1.** Properties of MATT and CF

| **Compound** | **Structure** | **Mw (g/mol)** | **Solubility** | **Formal Charge (mV)** |
| --- | --- | --- | --- | --- |
| MATT |  | 331.413 | slightly soluble | 0 |
| CF |  | 376.32 | slightly soluble | 0 |
